# Comparison of Frequency Bands Using Spectral Entropy for Epileptic Seizure Prediction

**DOI:** 10.1155/2013/287327

**Published:** 2013-05-25

**Authors:** Susana Blanco, Arturo Garay, Diego Coulombie

**Affiliations:** ^1^Dosimetry and Medical Equipment Laboratory UB, National Council for Scientific and Technological Research (CONICET), C1426DQG Buenos Aires, Argentina; ^2^Neurosciences Unit, Center for Medical Education and Clinical Research (CEMIC), C1431FWO Buenos Aires, Argentina; ^3^Department of Engineering and Technological Research, National University of La Matanza (UNLaM), B1754JEC San Justo, Argentina

## Abstract

*Introduction*. Under the hypothesis that the uncontrolled neuronal synchronization propagates recruiting more and more neurons, the aim is to detect its onset as early as possible by signal analysis. This synchronization is not noticeable just by looking at the EEG, so mathematical tools are needed for its identification. *Objective*. The aim of this study is to compare the results of spectral entropies calculated in different frequency bands of the EEG signals to decide which band may be a better tool to predict an epileptic seizure. *Materials and Methods*. Invasive ictal records were used. We measured the Fourier spectrum entropy of the electroencephalographic signals 4 to 32 minutes before the attack in low, medium and high frequencies. *Results*. The high-frequency band shows a markedly rate of increase of the entropy, with positive slopes and low correlation coefficient. The entropy rate of growth in the low-frequency band is practically zero, with a correlation around 0.2 and mostly positive slopes. The mid-frequency band showed both positive and negative slopes with low correlation. *Conclusions*. The entropy in the high frequencies could be predictor, because it shows changes in the previous moments of the attack. Its main problem is the variability, which makes it difficult to set the threshold that ensures an adequate prediction.

## 1. Introduction

Epilepsy is considered by some authors as a symptom that responds to different neurological disorders. These disorders may be strokes, head traumas, brain malformations, and the effect of neurotoxic substances among others [1]. All of them are discontinuities that affect normal brain activity, enabling the creation of conditions for the occurrence of the attack. The mechanism of the attack is not clear yet, although there are several hypotheses based on the incorrect communication between neurons [2]. For some reason, this results in synchronizing the activation of a mass of neurons which generates the crisis.

According to the more accepted postulates, the problem of the epilepsy occurs when an error in the communication between neurons is not blocked by the inhibition system that controls them. In this case one neuron excites another neuron causing a cascade of stimulation that grows and propagates recruiting other neurons.

If during that propagation neither factor inhibits the recruitment of neurons, that small initial failure takes a critical mass of neurons and ends in an epileptic seizure [3].

Under the hypothesis that the uncontrolled neuronal synchronization propagates and recruits more neurons each time [4], we are going to try to detect it as early as possible by signal analysis. This synchronization is not evident just by looking at the EEG, so we need mathematical tools to identify it. 

To deal with this problem, we propose to study the entropy of the signal, using it as a numerical method to evaluate the degree of freedom or dispersion of the spectral energy of the signal. We are going to divide the spectrum of the encephalographic signal into three bands. Then we calculate the variation of entropy for each spectral band.

This variation has the particularity to show, in a single number, the uniformity of the spectrum distribution. Higher entropy means that the energy of the spectrum is more distributed. Low entropy shows a greater concentration of energy in a group of band frequencies. The latter concept would permit to identify an eventual neuronal synchronization by modifying the spectrum.

This study aims to evaluate which of the bands is more sensitive to changes in the entropy of the signal before the attack occurs. It is assumed that, in brief periods before the attack, the entropy varies linearly. To evaluate the variation of entropy, we are going to obtain regression lines for each of the three bands. Also the correlation coefficient for each line is going to be calculated in order to observe the spread of values. Concerning the regression line we are going to consider its slope. This factor represents the entropy variation rate during the time. The band of higher rate is going to be the most sensitive. The one with less dispersion means greater certainty in the measurement.

## 2. Materials and Methods

### 2.1. Materials

We analyze the data of patient records from the “Epilepsy Center of the University Hospital of Freiburg” in Germany, used for “Freiburg seizure prediction project” [5], which were shared for experimental use.

The database contains records of EEG of 20 patients who suffer from focal epilepsy, pharmacologically intractable. Data were recorded during a presurgical invasive monitoring of epilepsy in the Epilepsy Center of the University Hospital of Freiburg in Germany. In order to obtain a high-ratio signal—noise, less artifacts and register directly from focal areas, grids, strips, subdural, and depth electrodes were used. EEG data were acquired using a digital EEG video system Neurofile NT with 128 channels, 256 Hz sampling frequency, and analog-digital conversion of 16 bits. Neither type of filter was used.

For each patient there exists a set of data called “ictal” which contains files with the seizure register at least 50 minutes before it happens. We used the focal channel record. The ages range from 14 to 50 years, 7 males and 13 females. The patients present different types of seizures (simple partial, complex, and generalized tonic-clonic), with different origin locations (frontal, temporal, occipital temporal, and parietal) and affection of different structures (hippocampus or neocortical).

### 2.2. Methods

To do the calculation, we used the spectral entropy (SEN), which is the Shannon entropy properly normalized and applied to the power spectrum density of the EEG signal. That is,

(1) $$\begin{array}{c} \text{SEN}=\frac{-\Sigma P_{k}\log_{}P_{k}}{\log_{}\left(N\right)}, \end{array}$$

where   *P*_*k*_ are the spectral powers of normalized frequency, such that   Σ*P*_*k*_ = 1 and *N* = number of frequencies (bin).

We analyzed the signal during the 4 to 32 minutes prior to the seizure. First the signals were filtered with a notch at 50 Hz (line filter in Germany) and then arbitrarily divided into bands, generating three vectors per patient. The filters used are of order 40 with Hamming window. The following are the frequencies for each band:

 Low band of frequencies: 0.1 to 12 Hz,
 Mid band of frequencies: 12 to 32 Hz,
 High band of frequencies: 32 to 128 Hz.

Each vector was analyzed with a time window for the application of the FFT of 16 s (4096 samples), without overlapping the windows. The frequency resolution was of 1024 steps (bin).

Once assumed the linear variation, we are going to obtain the regression lines and the correlation of the points representing each variation of the entropy. To improve the visualization and comprehension of the entropy variation, we are going to draw the movable mean with an averaging interval of 4 minutes.

## 3. Results 

**Figure 1:**
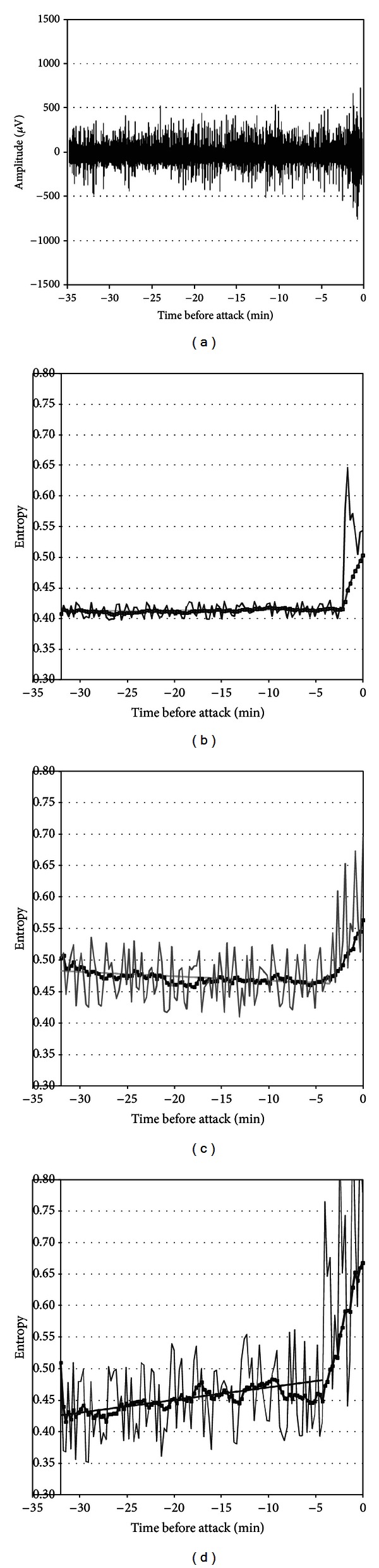
Example of a patient's analysis of entropy in bands. (a) EEG signal prior to the attack; (b) entropy analysis for low frequencies (continuous trace) with its regression line (continuous line) and its 4-minute moving average (dotted trace); (c) entropy for mid frequencies; (d) entropy for high frequencies.

**Figure 2:**
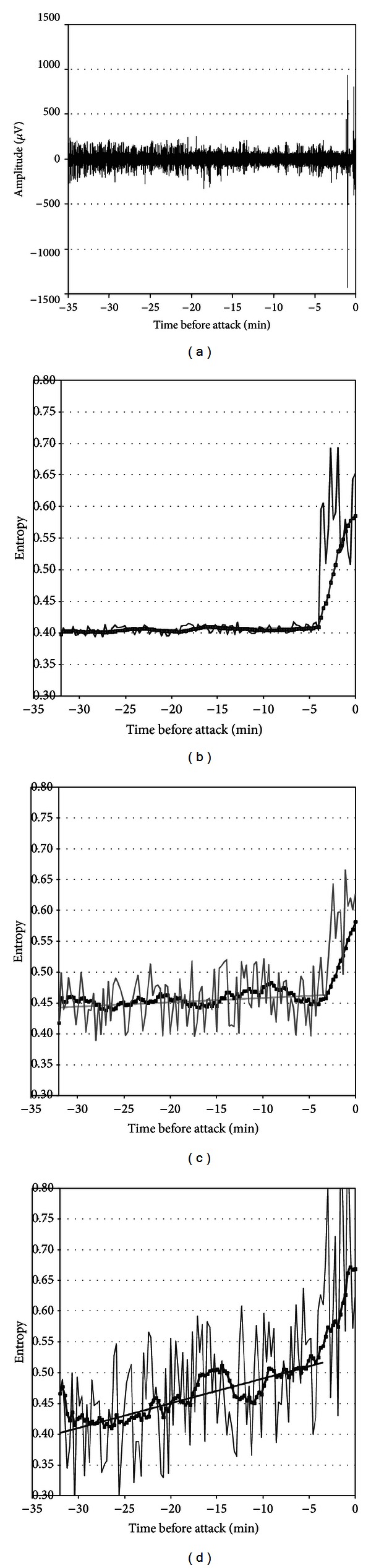
Another example of a patient's analysis of entropy in bands. (a) EEG signal prior to the attack; (b) entropy analysis for low frequencies (continuous trace) with its regression line (continuous line) and its 4-minute moving average (dotted trace); (c) entropy for mid frequencies; (d) entropy for high frequencies.

Figures 1 and 2 show the electroencephalographic signals and their respective entropy analyses for two patients with their regression lines in each band during the 30 minutes prior to the attack.

 These figures provide data to identify the entropy variation in function of time for each frequency band analyzed in the patient. The regression line and the correlations were calculated considering an interval of 32 to 4 minutes before the seizure. During the period between the previous 4 minutes and the seizure, the entropy can increase considerably, so we did not take it into account for the calculation.

Graphics show the entropy of this period very close to the attack, for illustrative purposes only. The graph corresponding to the moving average across the time of the entropy considers the whole period, from the previous 32 minutes to the time of the seizure.

Table 1 shows the slopes of the regression line and the correlation for each frequency band and each patient.

From the results we can identify that the highest band of frequencies shows a rate of entropy increase prior to the seizure that on the average for all the attacks is of *p* = 5.411 · 10^−3^, with a mean correlation coefficient *r* = 0.07. In all cases, the slopes are positive.

In the lowest band of frequencies, the mean rate of growth of the entropy is *p* = 1.77 · 10^−4^, with a correlation coefficient *r* = 0.2 in absolute value. Slopes are mostly positive, except in one sample that was negative.

In the band of the mid frequencies the rate of growth was *p* = 1.88 · 10^−4^, with a correlation coefficient *r* = 0.15 in absolute value. The slopes can be either positive or negative.

## 4. Discussion

The ability of mathematical methods of signal analysis in the recognition of nonevident changes for the visual detection becomes important for the observation of gamma activity. This activity has been observed in preictal animal records in EEG registers with implanted and external electrodes [6, 7]. A recent observation points out the importance of the detection of these rhythms in scalp registers for the detection of the focal crisis onset [8]. However the use of signal from scalp electrodes presents a disadvantage regarding the implanted electrodes. The first ones are often contaminated by EMG noise. This fact would produce an increase in the power of the fast frequencies, which is not observed in our analysis.

From the table of results we can see that the highest band of frequencies undergoes a more pronounced change of entropy as we approach the attack, evidenced by the slopes which are notoriously larger than in the other two bands. Instead, the correlation of values obtained with this line is very low, which shows us the dispersion of the entropy values when calculated for this band of frequencies. It is noteworthy that, in all the analyzed patients, the entropy increases in this band, meaning that its slope is positive.

In the opposite case, we have the lowest band of frequencies where there is a variation of entropy almost null and with a very superior correlation regarding the rest of the bands. 

The band of mid frequencies does not follow any fixed trend, changing both between positive and negative slopes and having low correlation. 

The change of entropy means that the spectrum energy is redistributing. The increase of the entropy evidences that a signal with energy concentrated in a band of frequencies tends to distribute that energy to the rest of the bands. The maximum entropy is achieved when all the bands have equal energy. In the band of the high frequencies, it could have a transference of energy from the low frequencies to the highest. A possible explanation would be to associate this redistribution of energy with communication faults between neurons, which occur at much higher frequencies than what is considered physiologically normal and which could trigger off the crisis process ending in the epileptic attack.

There exists an uncertainty factor due to the great variability of instantaneous entropy, which leads us to have a low correlation. This behavior could be associated with the communication errors, or on the contrary, to the action of some system of control which tries to inhibit the propagation of the error.

Recent findings perhaps could give us an alternative explanation because they show that, prior to the crisis, there exists desynchronization rather than synchronization, and the presence of gamma activity may then become an expression of columnar cortical fast spiking [9].

The spikes in the plot are another factor which contributes to the uncertainty of the entropy value. These spikes mostly stand out in the highest frequency band.

As a conclusion we can say that the entropy in high frequencies has great potential as a predictor because it reveals changes in the previous moments to the attack. Its main problem is the variability, which makes it difficult to setting the threshold that ensures an accurate prediction. This variability may be due to the presence of ubiquitous spikes in the electroencephalographic records of epileptic patients, that although they affect the whole spectrum, they are prevalent in the band of high frequencies. It could be a good criterion for future works to filter spikes and repeat the entropy measurement for the band of high frequencies. 

## Figures and Tables

**Table 1:** Regression line and correlation of each band of frequencies.

Overall results	Slopes (*p*)			Correlation (*r*) (absolute values)		
Patient	Low	Mid	High	Low	Mid	High
1	0.000116	0.000228	0.006246	0.20	0.14	0.09
2	0.000302	0.000453	0.006941	0.13	0.17	0.05
3	0.000271	−0.000766	0.007815	0.11	0.16	0.09
4	0.000205	−0.000639	0.008389	0.28	0.10	0.07
5	0.000254	0.000787	0.002268	0.24	0.11	0.07
6	0.000123	−0.000751	0.002566	0.22	0.14	0.08
7	0.000122	0.000574	0.004457	0.28	0.15	0.07
8	0.000118	0.000697	0.001109	0.16	0.14	0.07
9	0.000310	0.000111	0.004003	0.11	0.10	0.05
10	0.000175	0.000848	0.004635	0.20	0.14	0.10
11	0.000162	−0.000560	0.006605	0.27	0.08	0.10
12	0.000162	0.000315	0.008695	0.17	0.17	0.08
13	0.000306	0.000652	0.007029	0.19	0.16	0.08
14	0.000137	0.000112	0.009008	0.13	0.16	0.07
15	−0.000315	−0.000449	0.002743	0.13	0.18	0.07
16	0.000160	0.000843	0.009275	0.19	0.13	0.07
17	0.000313	0.000734	0.006408	0.28	0.12	0.05
18	0.000172	−0.000412	0.001995	0.21	0.17	0.05
19	0.000284	0.000120	0.002553	0.25	0.19	0.08
20	0.000168	0.000855	0.005470	0.31	0.19	0.07

Average	0.000177	0.000188	0.005411	0.20	0.15	0.07
